# The Prediction of Geocentric Corrections during Communication Link Outages in PPP

**DOI:** 10.3390/s20030602

**Published:** 2020-01-21

**Authors:** Joanna Janicka, Dariusz Tomaszewski, Jacek Rapinski, Marcin Jagoda, Miloslawa Rutkowska

**Affiliations:** 1 Institute of Geodesy, University of Warmia and Mazury in Olsztyn, Oczapowskiego str. 2, 10-719 Olsztyn, Poland; dariusz.tomaszewski@uwm.edu.pl (D.T.); jacek.rapinski@uwm.edu.pl (J.R.); 2 Faculty of Civil Engineering Environmental and Geodetic Sciences, Koszalin University of Technology, Sniadeckich 2, 75-453 Koszalin, Poland; marcin.jagoda@tu.koszalin.pl (M.J.); miloslawa.rutkowska@tu.koszalin.pl (M.R.)

**Keywords:** GNSS, SSR orbit corrections, prediction

## Abstract

The International GNSS Service (IGS) real-time service (RTS) provides access to real-time precise products. State-Space Representation (SSR) products are disseminated through the Internet using the Networked Transport of the RTCM (Radio Technical Commission for Maritime Services) via the Internet Protocol (NTRIP). However, communication outages caused by a loss of the communication link during ephemeris changes can occur. Unfortunately, any break in providing orbit and clock corrections affects the possibility to perform precise point positioning. To eliminate this problem, various methods have been developed and presented in the literature. The solution proposed by the authors is to directly predict geocentric corrections. This manuscript presents the results and analysis of geocentric correction predictions under two scenarios: the first between the IODE (issue of data ephemeris) value change and the second where prediction must be done for epochs containing a change in IODE ephemeris. In this case, the prediction uses data from a previous message. The Root Mean Square (RMS) values calculated based on the differences between the true correction values and the predicted geocentric corrections using a linear function, a second-degree polynomial and a constant value do not differ significantly. The numerical results show that, in most cases, maintaining the constant value of the last registered SSR correction is the best option.

## 1. Introduction

In the past few years, precise point positioning (PPP) has received a significant amount of attention in the GNSS community. PPP can be an effective option for precise real-time positioning, but it requires more information than GNSS observations alone. Reliable satellite orbit and clock corrections [1,2,3,4,5], ionosphere [6] and troposphere model parameters [7], and antenna phase center offsets and variations must be provided in order to obtain precise results [8]. In PPP, orbit and clock errors are of major importance. To provide maximum orbit accuracy in post processing PPP, precise orbit files (SP3) are usually used. Since ultra-rapid orbits and satellite clocks are available 3–9 h after observations, and final orbits are available 12–18 days after the observation time, this approach is not applicable to real time positioning.

The International GNSS Service (IGS) real-time service (RTS) provides real-time orbits and clock corrections to the broadcast ephemeris. These corrections are provided as real-time data-streams and are accessed via the internet using the Networked Transport of RTCM (Radio Technical Commission for Maritime) via the Internet Protocol (NTRIP). State-space representation (SSR) orbit and clock corrections are the most important real-time products used in real-time PPP [9]. Using broadcast ephemeris with SSR corrections results in a satellite position accuracy comparable to ultra-rapid SP3. In order to improve the positioning accuracy, SSR orbit corrections must be transmitted to PPP users every 1–2 min. Currently, real-time products are generated by different analysis centers: IGS (International GNSS Service), BKG (Bundesamt für Kartographie und Geodäsie), WUHAN (WUHAN University), ESA/ESOC (European Space Agency), CNES (Centre National d’Études Spatiales), DLR (Deutsches Zentrum Fur Luft und Raumfahrt), GMV (GMV Aerospace and Defense) and NRCan (Natural Resources Canada) using different algorithms. Despite this, the RTS product availability for the receiving user is lower than 100% because of possible communication link outages. Unfortunately, due to interruptions in the Internet connection, real-time positioning may suffer from an accuracy loss. A temporary loss of connection with the SSR stream provider can happen when the operator is using a cellular network far from urban areas or main routes [10]. In real-time applications, the correction data could be blocked due to poor signal reception [11]. Consequently, users can suffer from interruptions or delays in the transmission of their SSR corrections. Thus, any errors, outages, or delays may cause significant consequences for precise point positioning, especially for real-time applications. The subject of the bandwidth delay and the cost of data transfer for correction transmissions in the development of real-time PPP systems was widely discussed in [12,13].

One of the solutions to the problem of a temporary loss of connection with an SSR stream is to predict the orbital corrections during a disturbance. In [14], the concept of the short-time extrapolation of orbital correction terms from SSR orbit correction messages (radial, along, and cross-track) was introduced. The authors presented the accuracy of the predicted SSR corrections between each IODE (issue of data ephemeris) value change. Based on the research conducted so far in [14,15], in order to achieve the most accurate prediction, one should fit a polynomial to data at least three times longer than the prediction time. The results confirm that it is possible to forecast orbit correction parameters with a 1 cm accuracy for up to 5 min and with a 2 cm accuracy for up to 8 min [14]. In addition, the previous research results presented in [14,15] indicate that a second-degree polynomial provides satisfactory accuracy, while higher-degree polynomials do not provide a significant improvement.

Another solution to the problem of losing a communication link was presented by Gao [16], who suggested an extension of the existing SSR message package with additional solar radiation parameters (SRP) for satellite orbit and periodic satellite clock shift times [17].

SSR orbital corrections are defined as radial, along, and cross-track components, which consist of values and their rates of change. SSR orbital corrections are then transformed into geocentric corrections, and, finally, in order to obtain precise orbits, the geocentric corrections are applied to broadcast orbits.

This paper is focused on the issue of maintaining the best possible accuracy for the satellite position calculated from the broadcast ephemeris via the application of real-time corrections during the outage. We introduce geocentric orbit correction prediction as an alternative solution to the extrapolation of SSR orbit correction proposed by Hadaś and Bosy in [14]. In addition, the authors of [14] performed SSR orbit correction predictions only between IODE changes. They did not include the correction predictions over the time that the ephemeris changed.

We also investigate whether it is possible to predict the geocentric orbit correction values for a certain period of time if the time series of orbit corrections refer to outdated navigation messages.

If an interruption in the receiver–caster connection occurs, the user does not receive the SSR correction streams. The authors investigate if the extrapolation of geocentric corrections can allow one to extend the correction validity period without significantly degrading the solution in two scenarios:

- If a communication link outage occurs between the IODE value change;
- If a communication link outage occurs during the ephemeris change.

An outdated navigation message is understood as the situation when, after the change in the ephemeris, the SSR orbit and clock corrections are still calculated using the old ephemeris, and the position of the satellite is already calculated using the new ephemeris. Such situations occur at the moment of ephemeris change. Furthermore, sometimes a temporary loss of the communication link occurs, and the connection with the SSR correction streams is lost. This situation is interpreted by the authors as a “disturbance”

The prediction of corrections was made using three methods—linear function extrapolation, second-degree polynomial extrapolation, and maintaining the last registered correction value until the communication link is restored (later named the “constant value”). The software used to perform the tests is called PyGNSS and was created as part of a project implemented in cooperation with the manuscript authors. The PyGNNS software is used to perform WARTK (Wide Area Real-Time Kinematic) measurements. This software allows one to record various RTCM real-time streams to use them, for example, in algorithm testing. Among the other SSR correction streams, 1058, 1059, and 1060 can be recorded and replayed. Thus, the registered streams containing SSR orbit corrections were used to perform the tests. The results obtained in various test cases were compared to find the optimal method for geocentric correction prediction.

## 2. Satellite Orbit Corrections

Space-state representation aims to improve the broadcast orbits and clocks computed by the GNSS control segment to the accuracy of the precise ephemeris by providing satellite clock and orbit corrections. The procedures for utilizing SSR orbit messages in correcting broadcast orbits are presented in this section [14,18].

The corrections are calculated from the message reference time *t*_0_ to the current epoch *t* on the basis of the orbit correction *δO* values from the RTCM message:

(1) $$\;\delta O=\left[\begin{array}{c} \delta O_{r}\\ \delta O_{a}\\ \delta O_{c} \end{array}\right]+\left[\begin{array}{c} \delta {\overset{´}{O}}_{r}\\ \delta {\overset{´}{O}}_{a}\\ \delta {\overset{´}{O}}_{c} \end{array}\right]\left(t-t_{0}\right)$$

where *t* is the observation time; *t*_0_ is the reference time obtained from the SSR orbit correction message; *δO* = [*δO_r_*, *δO_a_*, *δO_c_*] are the orbit correction terms from the SSR orbit correction messages, radial, along, and cross-track, respectively; $\left[\delta \overset{´}{O_{r}},\;\delta {\overset{´}{O}}_{a},\;\delta {\overset{´}{O}}_{c}\right]$ are the rates of the orbit correction terms. The second step is to calculate the direction unit vector *e* in the radial *e_r_*, along-track *e_a_*, and cross-track *e_c_* directions:

(2) $$e_{r}=e_{a}\times e_{c}$$

(3) $$e_{a}=\frac{\overset{´}{r}}{\left|\overset{´}{r}\right|}$$

(4) $$e_{c}=\frac{r\times \overset{´}{r}}{\left|r\times \overset{´}{r}\right|}$$

where *r* is a satellite broadcast position, and vector $\overset{´}{r}$ is a satellite broadcast velocity vector.

The next step is to calculate the satellite position corrections *δP* (geocentric corrections) according to the following formula:

(5) $$\delta P=\left[\begin{array}{c} \delta X\\ \delta Y\\ \delta Z \end{array}\right]=\left[e_{r}e_{a}e_{c}\right]\delta O.$$

The satellite position computed from the broadcast ephemeris should be corrected by applying the geocentric corrections, *δP*. Then, the precise satellite position can be obtained:

(6) $$X_{P}=X_{broadcast}-\delta X\;Y_{P}=Y_{broadcast}-\delta Y\;Z_{P}=Z_{broadcast}-\delta Z.$$

**Figure 1:**
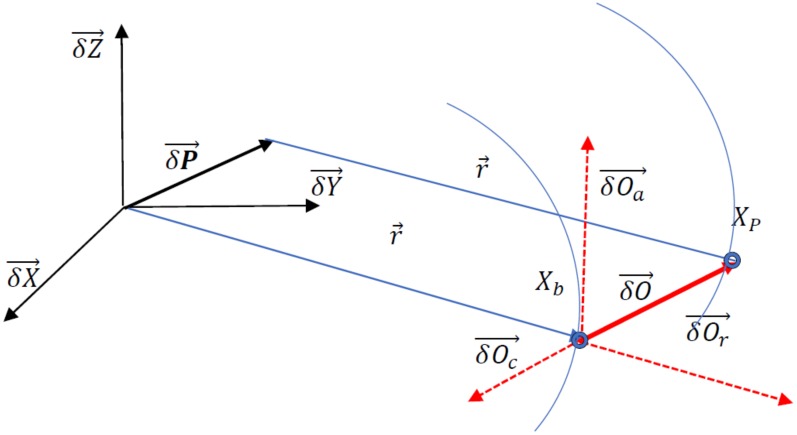
The interpretation of space-state representation (SSR) orbit corrections.

Figure 1 presents a geometrical interpretation of SSR orbit corrections.

## 3. Methods

The solution proposed in this paper is to directly predict geocentric corrections *δP* = [*δX*, *δY*, *δZ*] instead of extrapolating a radial and using along and cross-track SSR orbit corrections (as presented in [14]).

The tests were performed in two scenarios:

1. If the communication link outage occurs between the IODE value change;
2. If the communication link outage occurs during the IODE value change.

**Figure 2:**
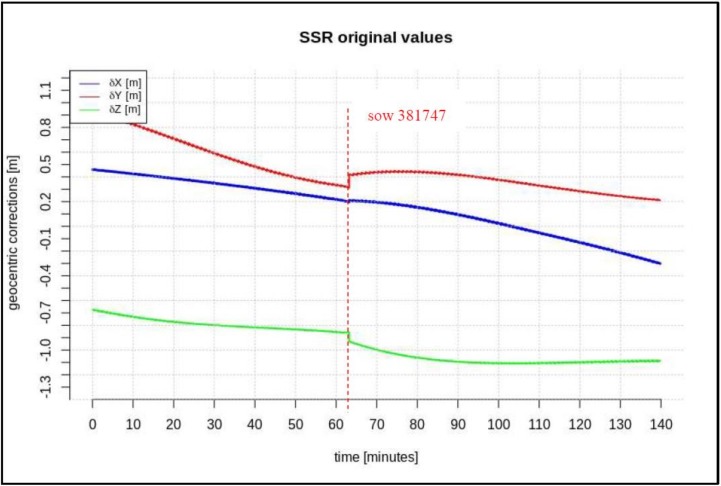
Time series of the geocentric corrections *δP* of DOY 99, GPS 13.

**Figure 3:**
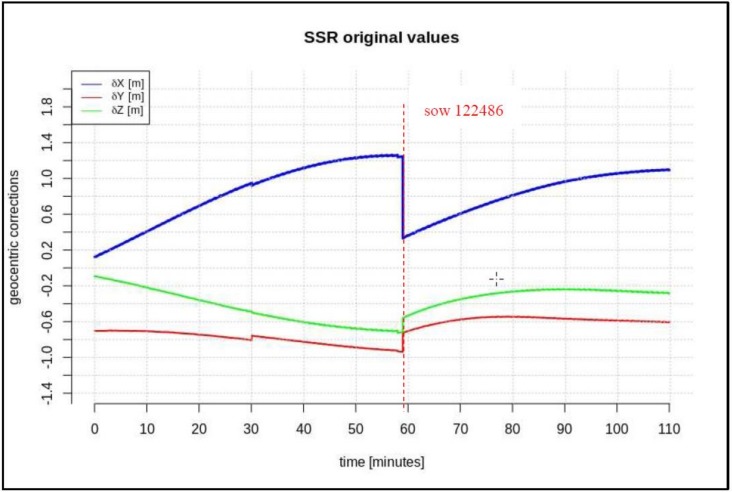
Time series of the geocentric corrections *δP* of DOY 85, GPS 28.

**Figure 4:**
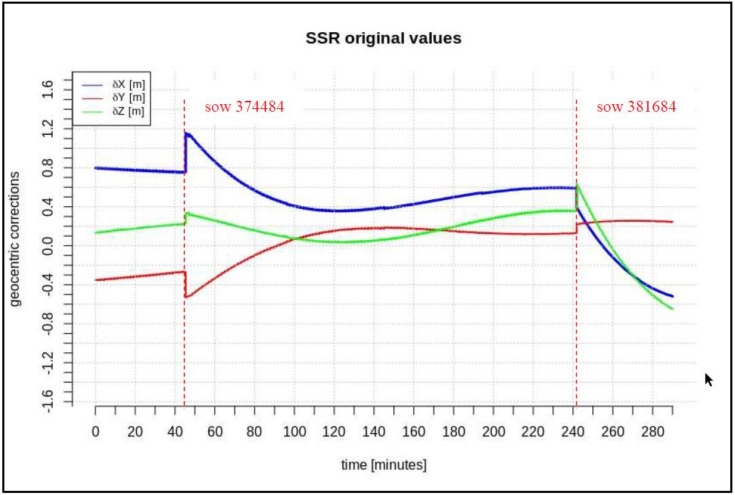
Time series of the geocentric corrections *δP* of DOY 102, GPS 30.

In addition, this paper describes the impact of the minimum required length of the data-stream on the short-term predictions of geocentric corrections and the maximum available data-stream in both scenarios. Based on the research presented in [14,15], one should fit the polynomial to data at least 3 times longer than the prediction time. After analyzing many correction streams for different satellites, it was found that the “disturbances” (marked as red lines in Figure 2, Figure 3 and Figure 4) last no longer than 2 min, so a short-term prediction period is required. It has been assumed that the prediction time of geocentric corrections will take about 5 min. Thus, the minimum required time for the data is 15 min. The maximum available data-stream refers to the time needed to form the registration start time to the communication link outage.

The predictions in both scenarios were performed using three approaches—a linear function, a second-degree polynomial, and a constant value (the last received SSR correction before the link outage). The linear function and a second-degree polynomial extrapolation were fit using the least square method, assuming that all of the observation weights were equal. The short-time prediction required several minutes.

Figure 2, Figure 3 and Figure 4 present the geocentric corrections calculated on the basis of the “original” SSR orbit corrections from the IGS service. The data-streams were recorded for three satellites: GPS 13, GPS 28, and GPS 30. In Figure 2, Figure 3 and Figure 4, one can also notice that the data are not continuous due to the IODE value changes (marked with red dashed lines in Figure 2, Figure 3 and Figure 4).

Figure 2, Figure 3 and Figure 4 show that the corrections may have different values, but they show a certain common feature—they look like fragments of unconnected polynomials.

The time span of pre-recorded SSR corrections is 100–280 min. The geocentric corrections were calculated according to Equations (1)–(6). In the next step, geocentric corrections, *δP*, were predicted for the two above-mentioned scenarios.

## 4. Results of Geocentric Correction Prediction

### 4.1. Geocentric Correction Extrapolation (GPS G13): The Communication Link Outage Occurs during the Ephemeris Change

**Figure 5:**
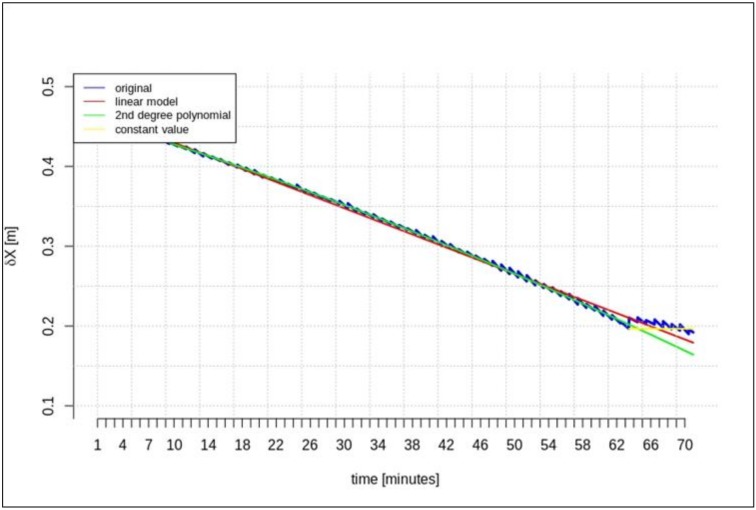
The results of the *δX* geocentric correction extrapolation (GPS G13) for a 70 min data-stream—the time series of orbit corrections refer to an out-of-date navigation message.

**Figure 6:**
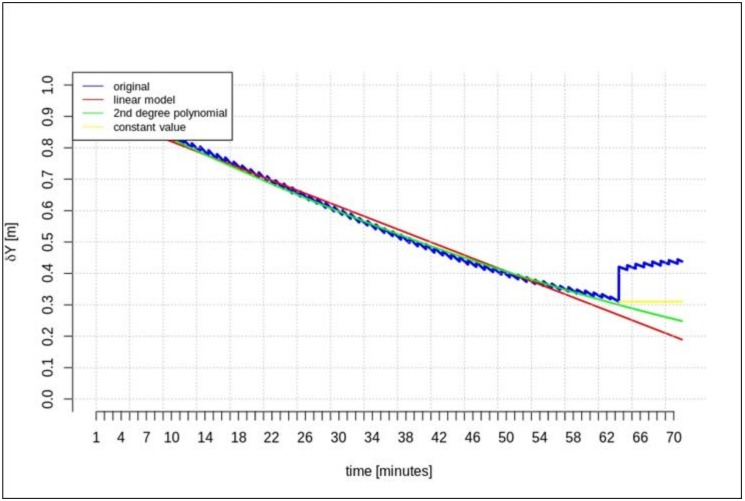
The results of the *δY* geocentric correction extrapolation (GPS G13) for a 70 min data-stream—the time series of the orbit corrections refer to an out-of-date navigation message.

**Figure 7:**
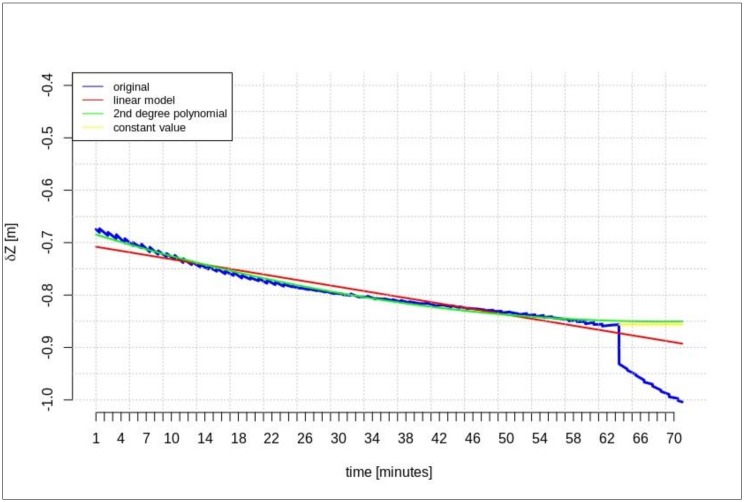
The results of the *δZ* geocentric correction extrapolation (GPS G13) for a 70 min data-stream—the time series of the orbit corrections refer to an out-of-date navigation message.

This section presents the results of the prediction using the time series of orbit corrections based on an outdated navigation message. Figure 5, Figure 6 and Figure 7 show the prediction results obtained on the basis of the calculations performed by different methods—linear prediction, second degree polynomial prediction, and constant value, respectively. The original geocentric corrections calculated based on the original SSR are presented in blue. The red and green lines represent the predicted values of the geocentric corrections with the use of the linear and polynomial methods, respectively. The yellow color shows the last received correction value, which is maintained until the communication link is recovered. An extrapolation was performed based on 65 min of data as the maximum registered time of the data-stream (from the start time to the communication link outage).

Figure 5, Figure 6 and Figure 7 show that at the 65th min of data registration, there was a change in the broadcast ephemeris, changing the values of the SSR corrections by several centimeters. Based on the 65 min data-stream, a 5-min prediction of geocentric corrections was performed, and the results are presented in Figure 5, Figure 6 and Figure 7.

Figure 5, Figure 6 and Figure 7 depict the results of orbital correction extrapolation using the three aforementioned methods. True corrections are plotted in blue. The differences between the true values and the predicted ones (regardless of the prediction method) are significant, while all three predictions look very similar.

**Figure 8:**
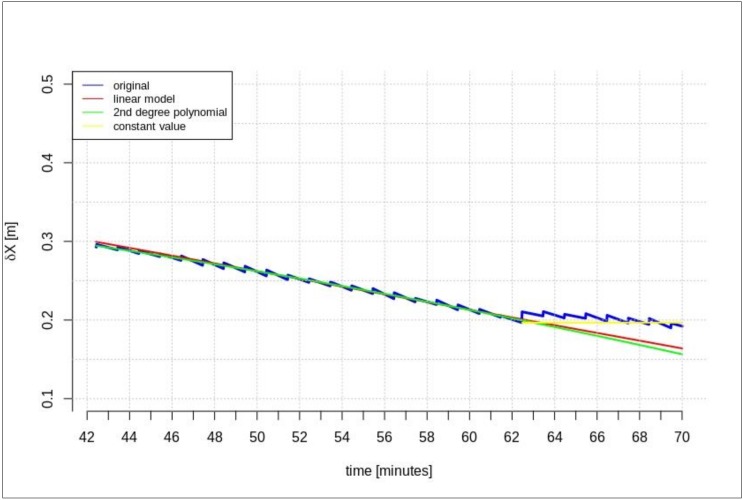
The results of the *δX* geocentric correction extrapolation (GPS G13) for a 15 min data-stream—The time series of the orbit corrections refer to an out-of-date navigation message.

**Figure 9:**
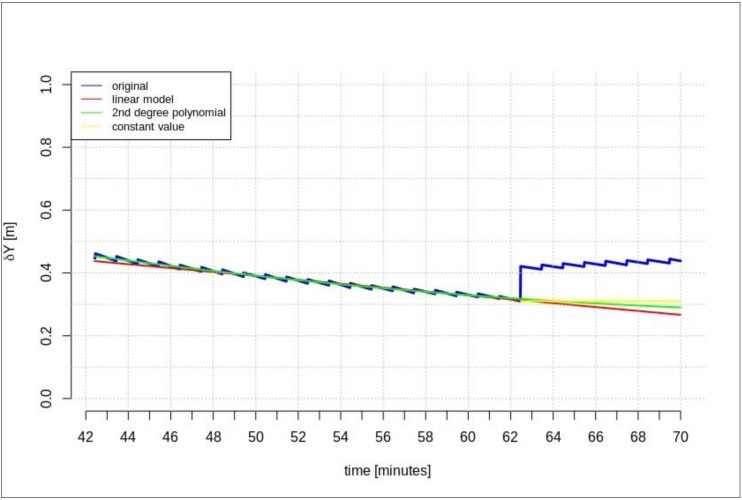
The results of the *δY* geocentric correction extrapolation (GPS G13) for a 15 min data-stream—The time series of the orbit corrections refer to an out-of-date navigation message.

**Figure 10:**
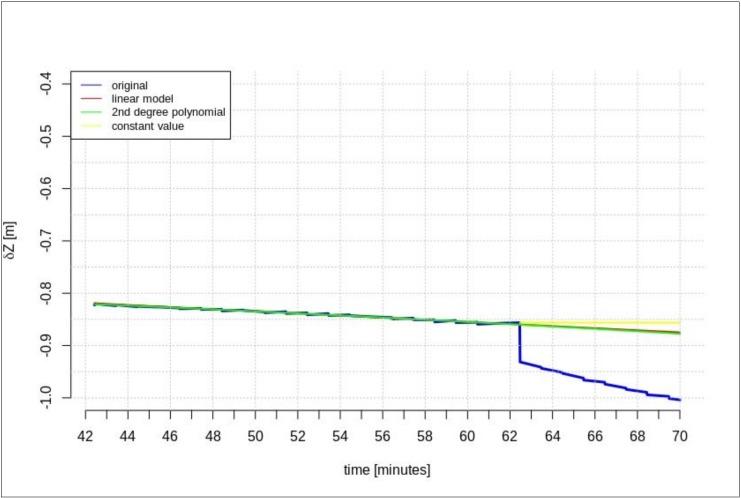
The results of the *δZ* geocentric corrections extrapolation (GPS G13) for a 15 min data-stream—the time series of the orbit corrections refer to an out-of-date navigation message.

The next test was performed based on a 15-min correction stream (which is the minimum required data-stream for a 5 min prediction). Figure 8, Figure 9 and Figure 10 show the results obtained in this scenario.

Figure 8, Figure 9 and Figure 10 show the significant difference between the original and predicted corrections and the small differences between the prediction methods. In Figure 10, the red and green lines overlap. 

Table 1 presents the RMS values calculated from the differences between the true correction values (the original SSR corrections) and the predicted geocentric corrections based on 65- and 15-min time periods. Linear 65, Polynomial 65, Linear 15, Polynomial 15, and constant value refer to 65-min, 15-min, and constant value data-streams, respectively.

The RMS values presented in Table 1 indicate that, if the time series of orbit corrections refer to an out-of-date navigation message, or if any disturbances occur, linear prediction or polynomials do not provide a better solution than maintaining the last registered value. The RMS values in Table 1 demonstrate that leaving the last registered values of the corrections is the best choice. As a part of this study, many data-streams were recorded, and many corrections were predicted for various satellites. Table 1 shows a selected example that is representative for all measured streams.

### 4.2. Geocentric Correction Extrapolation (GPS G28): The Communication Link Outage Occurs between the IODE Value Changes

**Figure 11:**
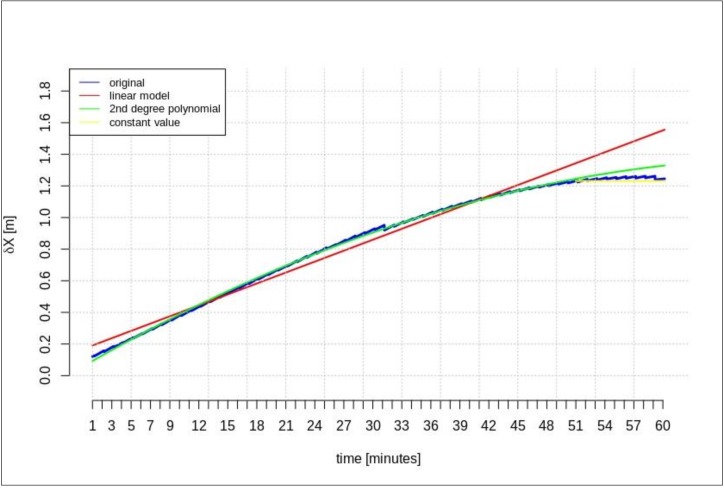
The results of the *δX* geocentric correction extrapolation (GPS G28) for a 60-min data-stream between each issue of data ephemeris (IODE).

**Figure 12:**
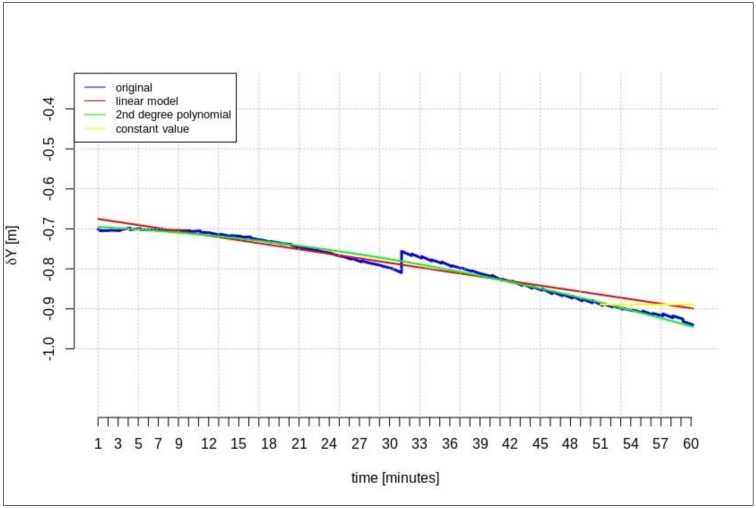
The results of *δY* geocentric correction extrapolation (GPS G28) for a 60-min data-stream between each IODE.

**Figure 13:**
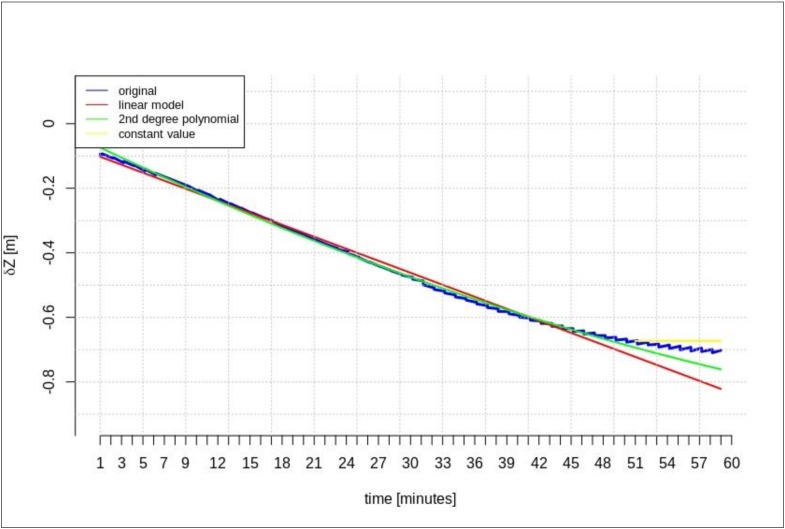
The results of the *δZ* geocentric correction extrapolation (GPS G28) for a 60-min data-stream between each IODE.

**Figure 14:**
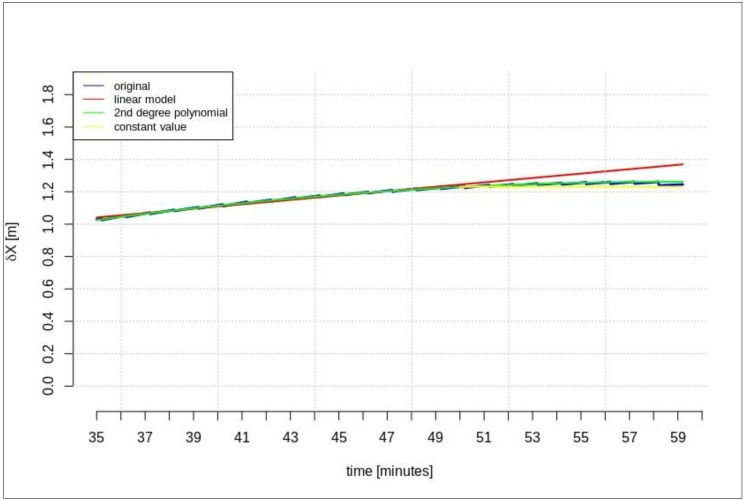
The results of the *δX* geocentric correction extrapolation (GPS G28) for a 15-min data-stream between each IODE.

**Figure 15:**
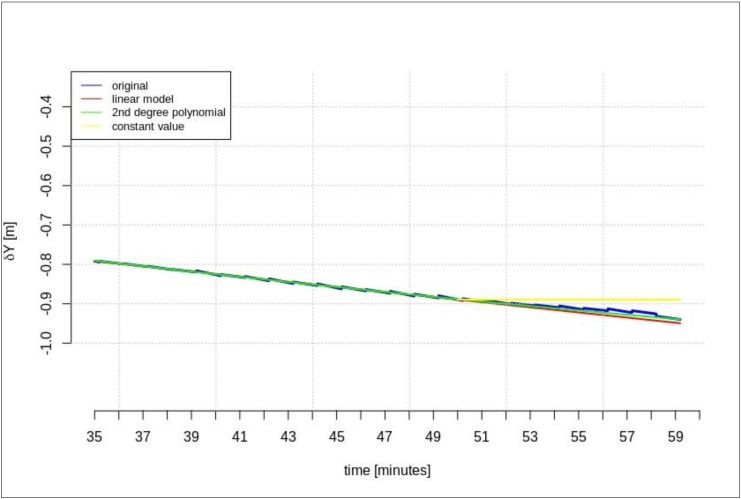
The results of the *δY* geocentric correction extrapolation (GPS G28) for a 15-min data-stream between each IODE.

**Figure 16:**
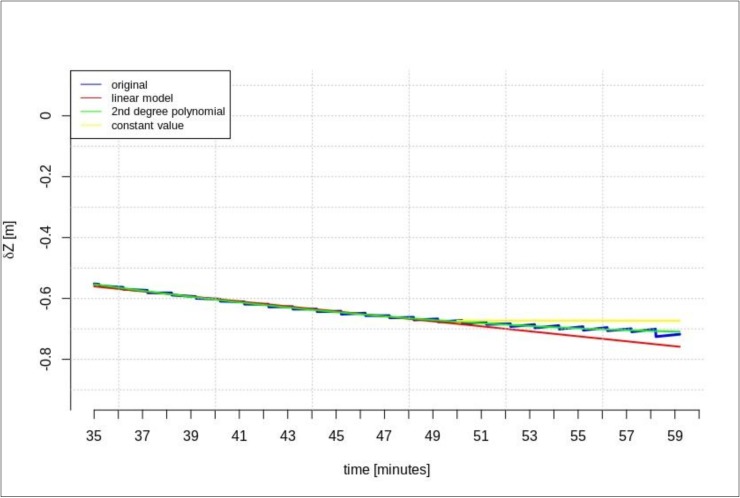
The results of the *δZ* geocentric correction extrapolation (GPS G28) for a 15-min data-stream between each IODE.

The next test was performed for the time series of orbit corrections between each IODE value change. According to the description included in the introduction, two cases of geocentric prediction were analyzed. In this section, the results of geocentric correction extrapolation are presented for a communication link outage occurring between the IODE value change. The calculations were also performed in two scenarios of data-stream length: the maximum and minimum required data-streams. The test was performed for the GPS G28 satellite, and the maximum recorded data-stream time was about 100 min (Figure 3). Figure 11, Figure 12, Figure 13, Figure 14, Figure 15 and Figure 16 show the extrapolation results.

The predicted values depicted in Figure 11, Figure 12 and Figure 13 vary depending on the prediction method. Unlike the scenario in Section 4.1, if the outage occurs between IODE changes, a worse result is found for linear interpolation. The results for the second degree polynomial and the last recorded value are similar.

Figure 14, Figure 15 and Figure 16 show the results for the minimum required stream length (15 min for a 5-min prediction). These results are also valid for the extrapolation between IODE changes.

The results obtained in this scenario confirm that the linear function, second-degree polynomial, and constant value provide similar values. Table 2 presents the RMS values calculated on the basis of the differences between the true correction values (the original SSR corrections) and the predicted geocentric corrections on the basis of a 50- and 15-min time period. The Linear 50, Polynomial 50, Linear 15, Polynomial 15, and constant value indicate 65-min, 15-min, and constant value data-streams, respectively

The same test was carried out for the GPS satellite G30. The range SSR was about 280 min for GPS 30. The resulting RMS values are summarized in Table 3 and Table 4.

The RMS values presented in Table 3 confirm that if the time series of orbit corrections refer to an out-of-date navigation message, the linear prediction or polynomial do not provide a better solution than retaining the last registered value (constant value). The results presented in Table 3 are related to another randomly selected satellite (GPS 30). The RMS values in Table 3 also demonstrate that leaving the last registered values for the corrections is the best choice.

The RMS values in Table 4 refer to GPS 30 *δX*, *δY*, and *δZ* corrections that predict if the time series of geocentric corrections are included between IODE value changes. In this case, the best solution is again to maintain the last registered value (the constant value) until communication link recovery.

### 4.3. Influence of Prediction Errors on PPP Results

The error budget in PPP consists of many factors, such as phase and code observation errors, ionosphere and troposphere modeling errors, and satellite and receiver clock errors. Errors in satellite position are not the biggest errors, but they can have a significant influence on the PPP results. In order to depict the influence of orbital errors alone on the PPP solution, a simulation was performed. The aim of this simulation was to visualize the relationship between orbital errors and the PPP solution without taking different error sources into account.

The following steps were performed:

- The coordinates of four satellites and one receiver were selected for a certain epoch;
- The satellite·receiver distances were calculated on the basis of their coordinates;
- A small amount of noise (max 1.5 cm) was added to all observations;
- The satellite coordinates were burdened with noise (a random walk) for 200 epochs;
- The receiver coordinates were calculated for each epoch using the minimum sum of the square satellite·receiver distance residuals as an objective function;
- The distance *D_i_* from the true coordinates to the coordinates obtained from the minimization of the objective function was calculated;
- A plot of the *dx_i_*, *dy_i_*, *dz_i_* increments of the satellite positions and *D_i_* was then created.

**Figure 17:**
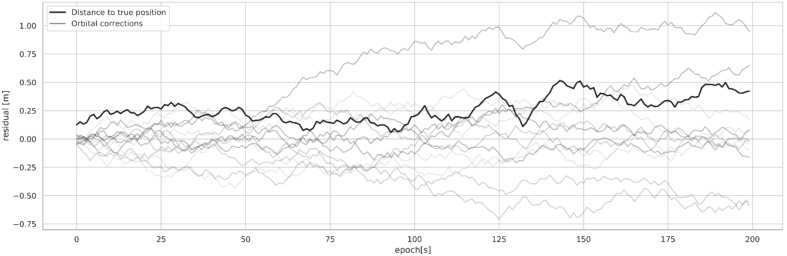
Influence of degrading orbital corrections on PPP results.

The increments are plotted with gray lines, and *D_i_* is plotted with a solid black line. The position accuracy degrades significantly with growing satellite coordinate increments. Figure 17 shows how the errors in geocentric corrections propagate to the final PPP solution when the correction accuracy degrades over time.

## 5. Discussion and Conclusions

This paper shows the possibility of short time (up to 5 min) geocentric correction (increment) predictions. Predicting increments is easier than predicting SSR orbit corrections, because it does not require one to convert orbital corrections to geocentric corrections. Therefore, it is not necessary to know the orbital parameters for a particular epoch. This whole procedure, then, is faster and simpler. Thus, even during a moment of interruption in the stream, the coordinates can be maintained based on a prediction of increments over a few minutes. Based on previous research, these predictions were performed with the use of a linear model, a second-degree polynomial and a constant value.

Significant changes in the geocentric correction values might be the result of an ephemeris change, but these changes could also be due to some other disturbances, such as the loss of a communication link. In this situation, a short-term prediction may be necessary until the communication link is recovered or the ephemerides are updated. Based on the tests performed in the two scenarios, one can assume that, in the case of the first scenario (the time series of orbit corrections referring to an out-of-date navigation message), the best solution is to maintain a constant value for a few minutes at the level of the last registered correction. Predicting corrections does not significantly affect the improvement of the position.

In the second scenario (Table 2), predictions using a second-degree polynomial with a 15 min data-stream allowed us to obtain lower RMS values than maintaining a constant value. However, the differences between the RMS for “Polynomial 15” and the “constant value” are about 2 cm, so both solutions are equally correct.

This conclusion relates with a limited amount of data (520 min of data from 3 GPS satellites) and shows that further research should be done to generalize the conclusions.

The simulation presented in Section 4.3 shows that the degradation of SSR correction accuracy in time can lead to significant PPP accuracy loss. SSR correction errors of magnitude 0 to ±0.77 m caused a significant PPP result drift. At the maximum, it was 0.76 m from the correct value.

## Figures and Tables

**Table 1:** The RMS values of the time series of geocentric corrections referring to an out-of-date navigation message (GPS G13).

	Linear 65 Maximum Data-Stream	Polynomial 65 Maximum Data-Stream	Linear 15 Minimum Data-Stream	Polynomial 15 Minimum Data-Stream	Constant Value
RMS_δX_ [m]	0.006	0.016	0.017	0.02	0.008
RMS_δY_ [m]	0.184	0.143	0.127	0.118	0.114
RMS_δZ_ [m]	0.079	0.108	0.093	0.092	0.102

**Table 2:** The RMS values of the time series of geocentric corrections between each IODE change value (GPS G28).

	Linear 50 Maximum Data-Stream	Polynomial 50 Maximum Data-Stream	Linear 15 Minimum Data-Stream	Polynomial 15 Minimum Data-Stream	Constant Value
RMS*_δX_* [m]	0.007	0.002	0.002	0.002	0.013
RMS*_δY_* [m]	0.031	0.022	0.012	0.004	0.019
RMS*_δZ_* [m]	0.019	0.011	0.001	0.001	0.019

**Table 3:** The RMS values of the time series of geocentric corrections, referring to an out-of-date navigation message (GPS G30).

	Linear Maximum Data-Stream	Polynomial Maximum Data-Stream	Linear 15 Minimum Data-Stream	Polynomial 15 Minimum Data-Stream	Constant Value
RMS*_δX_* [m]	0.166	0.418	0.348	0.338	0.342
RMS*_δY_* [m]	0.074	0.126	0.102	0.091	0.1
RMS*_δZ_* [m]	0.243	0.118	0.141	0.149	0.142

**Table 4:** The RMS values of the time series of geocentric corrections between each IODE change value (GPS G30).

	Linear Maximum Data-Stream	Polynomial Maximum Data-Stream	Linear 15 Minimum Data-Stream	Polynomial 15 Minimum Data-Stream	Constant Value
RMS*_δX_* [m]	0.211	0.035	0.019	0.004	0
RMS*_δY_* [m]	0.138	0.005	0.018	0.003	0
RMS*_δZ_* [m]	0.078	0.019	0.016	0.004	0.001
